# A Novel Radioimmune ^99m^Tc-Labeled Tracer for Imaging Sphingosine 1-Phosphate Receptor 1 in Tumor Xenografts: An *In Vitro* and *In Vivo* Study

**DOI:** 10.3389/fimmu.2021.660842

**Published:** 2021-08-18

**Authors:** Min Ye, Yongkang Gai, Hao Ji, Yaqun Jiang, Pengxin Qiao, Wenxia Wang, Yongxue Zhang, Xiaotian Xia, Xiaoli Lan

**Affiliations:** ^1^Department of Nuclear Medicine, Union Hospital, Tongji Medical College, Huazhong University of Science and Technology, Wuhan, China; ^2^ Hubei Province Key Laboratory of Molecular Imaging, Wuhan, China

**Keywords:** sphingosin-1-phosphate receptor 1, sphingosine-1 phosphate, ^99m^Tc-HYNIC-S1PR1mAb, radioimmune, SPECT, MCF-7 (breast cancer), SK-HEP-1

## Abstract

Sphingosine-1-phosphate (S1P) is a phospholipid that regulates pleiotropic biological activities and exerts extracellular functions by binding to five specific G-protein-coupled receptors, S1P receptors (S1PR) 1–5. When activated by S1P, S1PR promote the proliferation and invasion of tumor cells by inducing the formation of new blood vessels. We developed and assessed a new monoclonal antibody imaging probe ^99m^Tc-HYNIC-S1PR1mAb, to explore the feasibility of targeting the S1PR1 *in vitro* and *in vivo*. S1PR1mAb was prepared and followed by technetium-99m labeling with succinimidyl 6-hydraziniumnicotinate hydrochloride. Cell uptake and blocking studies were performed to investigate the binding specificity of ^99m^Tc-HYNIC-S1PR1mAb *in vitro*. ^99m^Tc-HYNIC-S1P1mAb was also tested *in vivo* in mice xenografted with SK-HEP-1 (high-expression of S1PR1) and MCF-7 (low-expression of S1PR1) using single-photon emission-computed tomography (SPECT). Ex vivo gamma counting of tissues from tumor-bearing mice was used to evaluate ^99m^Tc-HYNIC-S1PR1mAb biodistribution. The biodistribution study results showed significantly higher uptake in SK-HEP-1 tumors than in MCF-7 tumors (P < 0.001). Reduced uptake of ^99m^Tc-HYNIC-S1PR1mAb in SK-HEP-1 was observed in tumor-bearing nude mice pretreated with fingolimod, which binds competitively to the receptors, especially S1PR1. ^99m^Tc-HYNIC-S1PR1mAb can be synthesized and specifically targeted to S1PR1 *in vitro* and *in vivo*, allowing S1PR1 expression assessment with SPECT imaging.

## Introduction

Sphingosine-1 phosphate (S1P) is a bioactive sphingolipid metabolite that plays an important role in the maturation and homeostasis of the vascular system and in the transport of immune cells (1). S1P signaling pathways are involved in multiple biological processes, including tumor immune regulation, angiogenesis, tumor growth, tumor migration and invasion (2). which could be abnormal in breast cancer, liver cancer, melanoma, papillary thyroid cancer, pancreatic cancer, and prostate cancer (3).

S1P can bind to specific G protein-coupled receptors on the cell membrane surface to generate downstream signals that regulate physiological processes. The export of S1P is facilitated by the transporter protein Spinster homolog 2 or the ATP-binding cassette transporter protein family (4, 5). There are five known S1P receptor subtypes (S1PR1–5). S1PR1 is involved in, among many processes, promotion of proliferation and invasion of tumor cells and the formation of new blood vessels (6, 7). Studies have shown that S1PR1 is highly expressed in colorectal cancer and that the up-regulation of S1PR1 expression is closely related to deep infiltration of cancer cells and liver metastasis. Multivariate survival analysis has shown that S1PR1 expression levels can be used as an independent prognostic indicator of colorectal cancer (8). Among ER-positive breast cancer patients on tamoxifen therapy, patients with high S1PR1 expression had a higher treatment recurrence rate than those with low expression. Therefore, the expression of S1PR1 is promising as a biomarker to predict the drug resistance of tumors, to allow for more effective treatment (9). Further studies have indicated elevated expression of S1PR1 in bladder cancer tissue. S1PR1 stimulates bladder cancer cells to secrete transforming growth factor (TGF)-β and IL-6, thereby inducing the aggregation of regulatory T-cells (Tregs) (10). These studies demonstrate that S1PR1 is a promising cancer biomarker that may play a role in the prognosis of certain tumors.

Some studies have used S1PR1 as a therapeutic target for tumors. The combination of S1PR1 antagonist and chemotherapy drugs can be used as a new strategy for tumor treatment (11). In addition, for the S1PR1 signal is necessary in vascular stability, the loss of S1PR1 function will produce disordered and non-functioning blood vessels and increase the permeability of the blood vessels. Inhibition of S1PR1 can destroy tumor blood vessels in xenograft tumor models and ultimately inhibit tumor growth (12). In vivo studies have shown that S1PR1 inhibitors can reduce the vascular stability of Lewis lung cancer and inhibit angiogenesis and tumor growth (13). On the other hand, the vascular endothelial growth factor (VEGF) can promote tumor angiogenesis and tumor growth (14), and simultaneous inhibition of S1PR1 and VEGFR can maximize the effect of anti-angiogenesis therapy and may become an effective treatment strategy for the treatment of renal cell carcinoma and other types of tumors (15).

In view of the important role of S1PR1 in the occurrence, development and metastasis of tumors, noninvasive monitoring of the expression of S1PR1 in malignant tumors has important clinical significance in formulating tumor treatment plans, monitoring treatment effects, and evaluating prognosis. In some recent studies, Tu’s and Haufe’s groups have designed and synthesized a series of S1PRs, including specific carbon-11- and fluorine-18-labeled radiotracers highly targeted to S1PR1 (16–21). They have shown promising cell membrane binding assays *in vitro* towards S1PRs. Some *in vivo* evaluation of these analogs was accomplished. Single-photon emission computed tomography (SPECT) radioactive probes targeting S1PR1 are rarely known (22, 23). In this study, the S1PR1-targeted monoclonal antibody (S1PR1mAb), which can specifically target the S1PR1 protein on the cell surface, was designed and synthesized. The antibody was radiolabeled with technetium-99m using succinimidyl 6-hydraziniumnicotinate hydrochloride (SHNH, Solulink, Inc., San Diego, CA, USA). The targeting efficiency and pharmacokinetics of ^99m^Tc-labeled S1PR1mAb were then assessed to explore its potential clinical value in both *in vitro* and *in vivo* studies.

## Methods and Materials

### Antibody Preparation

We searched the human S1PR1 receptor protein sequence in the protein database of the National Center for Biotechnology Information (NCBI). The production and purification of the murine monoclonal antibody was done by ChinaPeptides Co., Ltd.

### Cell Culture

The human hepatocellular carcinoma cell line SK-HEP-1 (high expression of S1PR1) and the human breast carcinoma cell line MCF-7 (low expression of S1PR1) were purchased from the Type Culture Collection of the Chinese Academy of Sciences, Shanghai, China (CAT# TCHu109 and TCHu74). SK-HEP-1 cells were cultured in Minimum Essential Medium (MEM, Gibco, USA) and MCF-7 cells were cultured in Dulbecco’s Modified Eagle’s Medium (DMEM, Gibco, USA). Both medias were supplemented with 10% fetal bovine serum (FBS, HyClone, USA), 100 U/ml penicillin and 100 μg/mL streptomycin (Solarbio, Shanghai, China). The *cultures* were maintained at 37°C in 5% CO_2_ *incubator*s.

### Western Blot Analysis

When SK-HEP-1 and MCF-7 cells grew to a density of about 80%, we collected them and added cell lysate buffer (Beyotime, Shanghai, China) and isolated the protein. The protein concentration was determined using a bicinchoninic acid (BCA) protein assay kit (Boster, Wuhan, China). After SDS–PAGE electrophoresis, proteins were transferred to PVDF membrane. The membrane was incubated with the anti-S1PR1 antibody (Abcam, ab233386) for one night and then an anti-rabbit secondary antibody (Sanjian, Tianjin, China, LK2001) for 2 h. Images were processed with Image J software.

### Monoclonal Antibody Labeling and Purification

The procedure for S1PR1mAb radiolabeling comprised two key steps. First, SHNH (40 μg, 140 nmol, Beijing Bailingwei Technology Co., Ltd.) was added to the S1PR1mAb (140 μL, 1 nmol, 7.1nmol/mL) and reacted in darkness overnight at 4°C. After filtering, 100 μL tricine (100 mg/mL, Sigma-Aldrich, USA), 4μL SnCl_2_ (7 mg/mL, Sigma-Aldrich) and ${\;}^{99\text{m}}\text{Tc}{\text{O}}_{4}^{-}$ (900-1100mBq, 25-30 mCi, Beijing Atom High Tech, Beijing, China) were added to the reactions and incubated for 30 min at room temperature to prepare ^99m^Tc-HYNIC-S1PR1mAb.

The labeled compound was then purified on a PD-10 column (General Electric, Milwaukee WI, USA). The radiolabeled compound was analyzed by instant thin layer chromatography (ITLC) to calculate its radiolabeling efficiency, radiochemical purity, and *in vitro* stability (3, 6, 9, and 12 h in FBS, n = 4 per group). Fifty percent acetonitrile and 0.01M PBS were used as the developing solvent system.

### Cell Uptake Assays

^99m^Tc-HYNIC-S1PR1mAb uptake assays were performed in the SK-HEP-1 and MCF-7 cell lines. Briefly, the cells were plated in 24-well plates (1.5 × 10^5^ cells/well) and then incubated with 800 μL serum-free DMEM, or MEM containing ^99m^Tc-HYNIC-S1PR1mAb (37 kBq, 1μCi) at 37°C for 0.5, 1, 2, 4 and 6 h, respectively. The cells were then rinsed twice with 1 mL PBS and lysed with 1 N NaOH. The cell lysates *and supernatants* were collected. Radioactivity was measured with an automatic well-type gamma counter (2470,WIZARD^®^; PerkinElmer, Waltham, MA, USA). For blocking studies, SK-HEP-1 and MCF-7 cells were incubated with fingolimod (FTY720, Yuanye, Shanghai, China) (1 nmol) at 37°C for 4 h prior to experiments.

### Tumor Models

All animal experiments were performed in accordance with a protocol approved by the Institutional Animal Care and Use Committee of Tongji Medical College, Huazhong University of Science and Technology. Female BALB/c nude mice (3-4 weeks old, weighing 15.0-17.0g) were obtained from the *Huafukang* Bio-Technology *Company* (Beijing, China). SK-HEP-1 or MCF-7 cells in the logarithmic growth phase were inoculated subcutaneously into the right axilla with 1 × 10^6^ cells per mouse. The mice were used as models for *in vivo* SPECT imaging and biodistribution studies when the tumor size reached 0.5-1.0 cm.

### SPECT Imaging and Biodistribution

The SK-HEP-1 and MCF-7 tumor-bearing nude mice were injected with 37 MBq (37.0MBq, 1mCi) of ^99m^Tc-HYNIC-S1PR1mAb by tail vein. Static imaging was performed using SPECT with a pinhole collimator (Symbia T6^®^; Siemens, Erlangen, Germany) at 2, 6, 12, 18, and 24 h after injection, with corresponding acquisition times of 5, 10, 15, 20 and 30 min. Each anesthetized mouse was placed on the scanner table in the prone position. For the blocking study, SK-HEP-1 tumor-bearing nude mice received intragastric FTY720 (3 mg/kg/d) for 1 week before imaging.

For biodistribution, the SK-HEP-1 and MCF-7 tumor-bearing nude mice were injected with 29.6 MBq (29.6 MBq, 0.8 mCi) of ^99m^Tc-HYNIC-S1PR1mAb by tail vein injection with the blocking group of SK-HEP-1 xenografted mice having received intragastric fingolimod (3 mg/kg/d) for 1 week prior. After the mice were sacrificed by cervical dislocation, blood, brain, lung, heart, liver, spleen, kidney, stomach, small intestine, large intestine, muscle, bone, tail and tumor tissue were taken out and measured using an automatic gamma counter after washing and weighing. After attenuation correction, the percentage of injected dose per gram of tissue (%ID/g) was calculated for each tissue type and the radioactivity count ratio of tumor-to-blood (T/B) and tumor-to-muscle (T/M) of each tumor-bearing mouse were calculated.

### Tissue S1PR1 Expression Levels

For immunohistochemistry, SK-HEP-1 and MCF-7 tumor-bearing nude mice were sacrificed by cervical dislocation after SPECT imaging. Lung, liver, spleen, kidney, muscle and tumor tissues were removed and fixed with 4% paraformaldehyde. Subsequent tissue staining was performed by *Biossci* Biotechnology (Hubei, China). For western blotting, appropriate amounts of SK-HEP-1 and MCF-7 tumor tissues were rinsed with PBS 2–3 times, and then minced with scissors. Tissues were lysed in *radioimmune precipitation* buffer supplemented with a protease inhibitor mixture for 30 min at 4°C, and centrifuged for 10 min (4°C, 10,000 rpm). The supernatant was considered to comprise the *total protein extracts*, and then followed the remaining steps with the western blot of the cell proteins.

### Statistical Analysis

Statistical analysis was performed using Statistical Package for the Social Sciences (SPSS) software (version 22.0, SPSS Inc., Chicago, IL, USA). All data are expressed as mean ± standard deviation (SD). Differences were considered statistically significant when P values were < 0.05.

## Results

### S1PR1 Expression *In Vitro*

**Figure 1:**
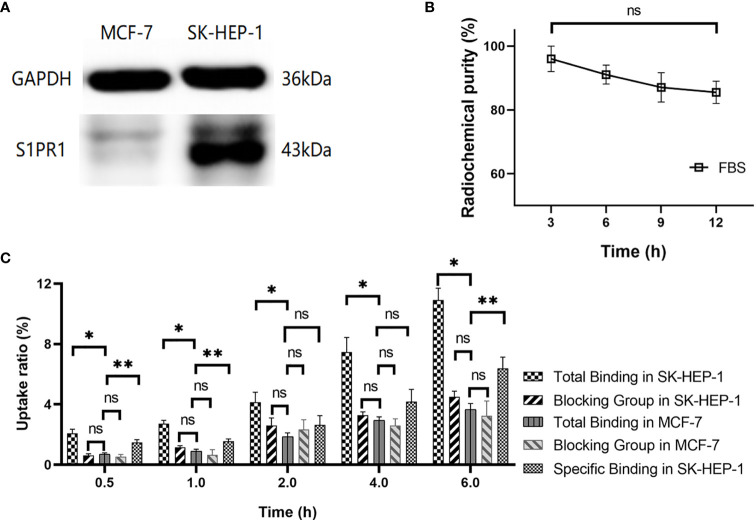
**(A)** Western blot analysis of S1PR1 expression in SK-HEP-1 cells and MCF-7 cells; **(B)** Stability of ^99m^Tc-HYNIC-S1PR1mAb *in vitro* at 3h, 6h, 9h, 12h in FBS; **(C)** *In vitro* cellular uptake. The total bindings and blocking results of ^99^mTc-HYNIC-S1PR1mAb in SK-HEP-1 cells (experiment group) and MCF-7 cells (control group) were included in panel C, as well as the specific binding in SK-HEP-1 cells. There was no significant difference between the blocking and non-blocking in MCF-7 cells. *, **, and ns indicate P < 0.05 and P > 0.05 (n = 4), respectively.

Western blotting was used to analyze the expression of S1PR1 in SK-HEP-1 and MCF-7 cells. The results demonstrated that SK-HEP-1 cells overexpressed S1PR1, while MCF-7 cells rarely expressed S1PR1 (**Figure 1A** and **Supplementary Figures 2**–**4**).

### Radiochemical Characteristics of ^99m^Tc-HYNIC-S1PR1mAb

The radiolabeling yield of ^99m^Tc-HYNIC-S1PR1mAb was 61.45 ± 9.16% (four assays). After purification on a PD-10 column, the radiochemical purity was 96.70 ± 0.04% (four separate purifications), which indicated high radiochemical purity (**Supplementary Figure 1**). The specific activity of ^99m^Tc-HYNIC-S1PR1mAb is 4.4MBq/μg. The purified ^99m^Tc-HYNIC-S1PR1mAb was placed in FBS and incubated at 37°C for *in vitro* stability analysis. The results showed that the stability of the molecular probes in FBS were 95.99 ± 3.99%, 91.05 ± 2.96%, 87.06 ± 4.58%, and 85.49 ± 3.48% at 3, 6, 9, and 12 h, respectively (**Figure 1B**). The difference in the stability of ^99m^Tc-HYNIC-S1PR1mAb at 3h and 12h was not statistically significant (P = 0.17).

### Cell Uptake Study

As shown in **Figure 1C**, the uptake rate of ^99m^Tc-HYNIC-S1PR1mAb by SK-HEP-1 cells (experimental group) increased significantly with time, the 0.5 h uptake rate was 2.08 ± 0.27%, and the 6 h uptake rate was 10.90 ± 0.79% (four separate measurements). The uptake rate in the blocking group of SK-HEP-1 cells, incubated with 0.1µM FTY720 4 h earlier, was significantly lower, measuring 0.62 ± 0.11% at 0.5 h and 4.51 ± 0.36% (n = 4) at 6 h. The specific binding of ^99m^Tc-HYNIC-S1PR1mAb in SK-HEP-1 cells were 1.47 ± 0.19% at 0.5h, 1.55 ± 0.16% at 1h, 2.64 ± 0.61% at 2h, 4.18 ± 0.81% at 4h and 6.39 ± 0.74% at 6h. The uptake rate in MCF-7 cells was similar to the blocking group in the SK-HEP-1, measuring 0.69 ± 0.11% at 0.5 h and 3.96 ± 0.39% (n = 4) at 6 h (P > 0.05). There was no significant difference between the blocking and non-blocking in MCF-7 cells (3.24 ± 0.98 *vs*. 3.66 ± 0.39%, 6h, P > 0.05) (**Figure 1C**).

### SPECT Imaging

**Figure 2:**
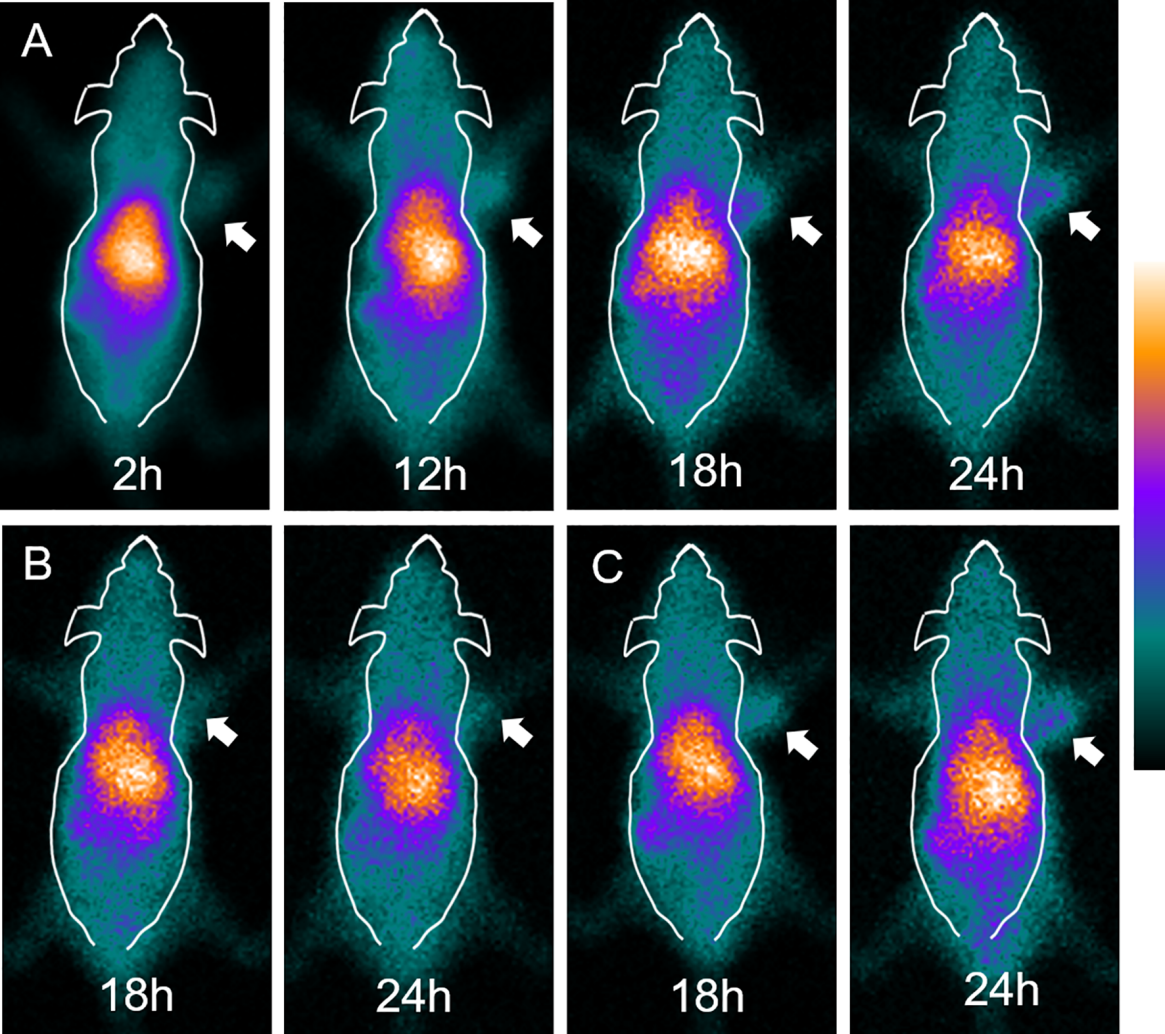
SPECT imaging of tumor-bearing nude mice injected with ^99m^Tc-HYNIC-S1PR1mAb. **(A)** SPECT imaging of SK-HEP-1 tumor-bearing nude mice at 2 h, 12 h, 18 h and 24 h. **(B)** SPECT imaging of blocked SK-HEP-1 tumor-bearing nude mice at 18 h and 24 h. **(C)** SPECT imaging of MCF-7 tumor-bearing nude mice at 18 h and 24 h The white arrows indicate the tumor site.

SPECT imaging results of SK-HEP-1 tumor model mice at 2, 12, 18, and 24 h are shown in **Figure 2A**. After injection of ^99m^Tc-HYNIC-S1PR1mAb, the uptake in SK-HEP-1 tumor-bearing nude mice was mainly in the liver. The uptake at the tumor site gradually increased with time, with tumors becoming clear at 18 h and 24 h. The SK-HEP-1 tumor xenografts in the blocking SK-HEP-1 tumor model group (**Figure 2B**) and in the MCF-7 xenografts (**Figure 2C**) in the control group were close to the background level at 2 h, and no obvious further increase in uptake occurred. At 24 h, the uptake of the imaging agent by tumors in the blocking group and the control group was significantly lower than that in the experimental group.

### Biodistribution Studies

**Figure 3:**
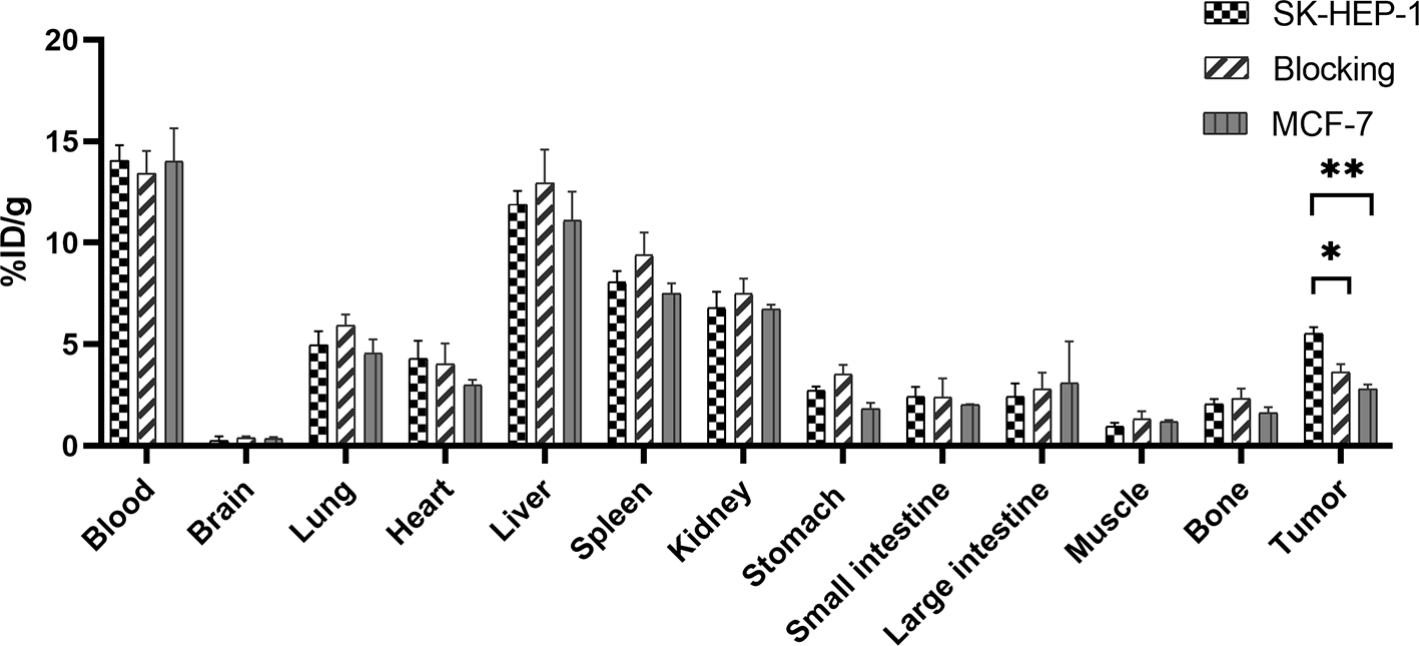
Biodistribution study of ^99m^Tc-HYNIC-S1PR1mAb in SK-HEP-1 tumor-bearing nude mice, blocked SK-HEP-1 tumor-bearing nude mice and MCF-7 tumor-bearing nude mice at 24 h post-injection. * and **P < 0.05 (n = 4).

To further explore the metabolism and specificity of ^99m^Tc-HYNIC-S1PR1mAb *in vivo*, biodistribution studies were performed on tumor-bearing nude mice in the SK-HEP-1 xenografted mice group, the blocking group (SK-HEP-1 xenografted mice treated with FTY720) and the MCF-7 xenografted mice. The results are shown in **Figure 3** and **Table 1**. When ^99m^Tc-HYNIC-S1PR1mAb was injected into the SK-HEP-1 xenografted mice group *via* the tail vein for 24 h, the probes were mainly concentrated in the blood and taken up by the liver, spleen and kidneys, indicated that the blood pool activity was slow to clear. The uptake values of the xenografted nude mice in blood, liver, spleen and kidney were 14.06 ± 0.75%ID/g, 11.89 ± 0.67%ID/g, 8.06 ± 0.54%ID/g, 6.79 ± 0.81%ID/g, respectively (n = 4). At 24 h after the injection of ^99m^Tc-HYNIC-S1PR1mAb, the SK-HEP-1 tumor uptake was 5.53 ± 0.32%ID/g, which was higher than the tumor uptake values of the tumor-bearing nude mice in the blocking group (P = 0.001) and MCF-7 xenografted mice group (3.64 ± 0.39%ID/g, 2.81 ± 0.21%ID/g, respectively)(P < 0.001). At the same time, the tumor/blood and tumor/muscle ratios of the tumor-bearing nude mice in the experimental group were higher than those in the blocking group and the control group. The biodistribution results were consistent with the SPECT imaging results.

**Table 1:** Biodistribution of ^99m^Tc-HYNIC-S1PR1mAb in SK-HEP-1 (including the SK-HEP-1 group and the blocking group) and MCF-7 tumor-bearing nude mice at 24 h post-injection.

Organs	SK-HEP-1	Blocking	MCF-7
**Blood**	14.06 ± 0.75	13.43 ± 1.10	14.03 ± 1.61
**Brain**	0.38 ± 0.04	0.39 ± 0.09	0.36 ± 0.09
**Lung**	4.98 ± 0.67	5.92 ± 0.55	4.56 ± 0.68
**Heart**	4.29 ± 0.88	4.03 ± 1.01	3.01 ± 0.24
**Liver**	11.89 ± 0.67	12.96 ± 1.64	11.09 ± 1.44
**Spleen**	8.06 ± 0.54	9.40 ± 1.10	7.51 ± 0.50
**Kidney**	6.79 ± 0.81	7.49 ± 0.76	6.72 ± 0.24
**Stomach**	2.72 ± 0.21	3.52 ± 0.47	1.82 ± 0.30
**Small intestine**	2.43 ± 0.47	2.39 ± 0.93	2.03 ± 0.03
**Large intestine**	2.42 ± 0.66	2.82 ± 0.79	3.11 ± 2.03
**Muscle**	0.95 ± 0.20	1.33 ± 0.38	3.11 ± 2.03
**Bone**	2.05 ± 0.26	2.34 ± 0.49	1.62 ± 0.29
**Tumor**	5.53 ± 0.32	3.64 ± 0.39	2.81 ± 0.21
**Uptake ratio**			
**Tumor/Blood**	0.39 ± 0.01	0.27 ± 0.04	0.21 ± 0.02
**Tumor/Muscle**	5.99 ± 1.38	2.84 ± 0.52	2.38 ± 0.07

All data were expressed as mean ± SD. The uptake in each tissue was expressed as the percentage of injected dose per gram of tissue (%ID/g) (n=4).

### Tissue S1PR1 Expression

**Figure 4:**
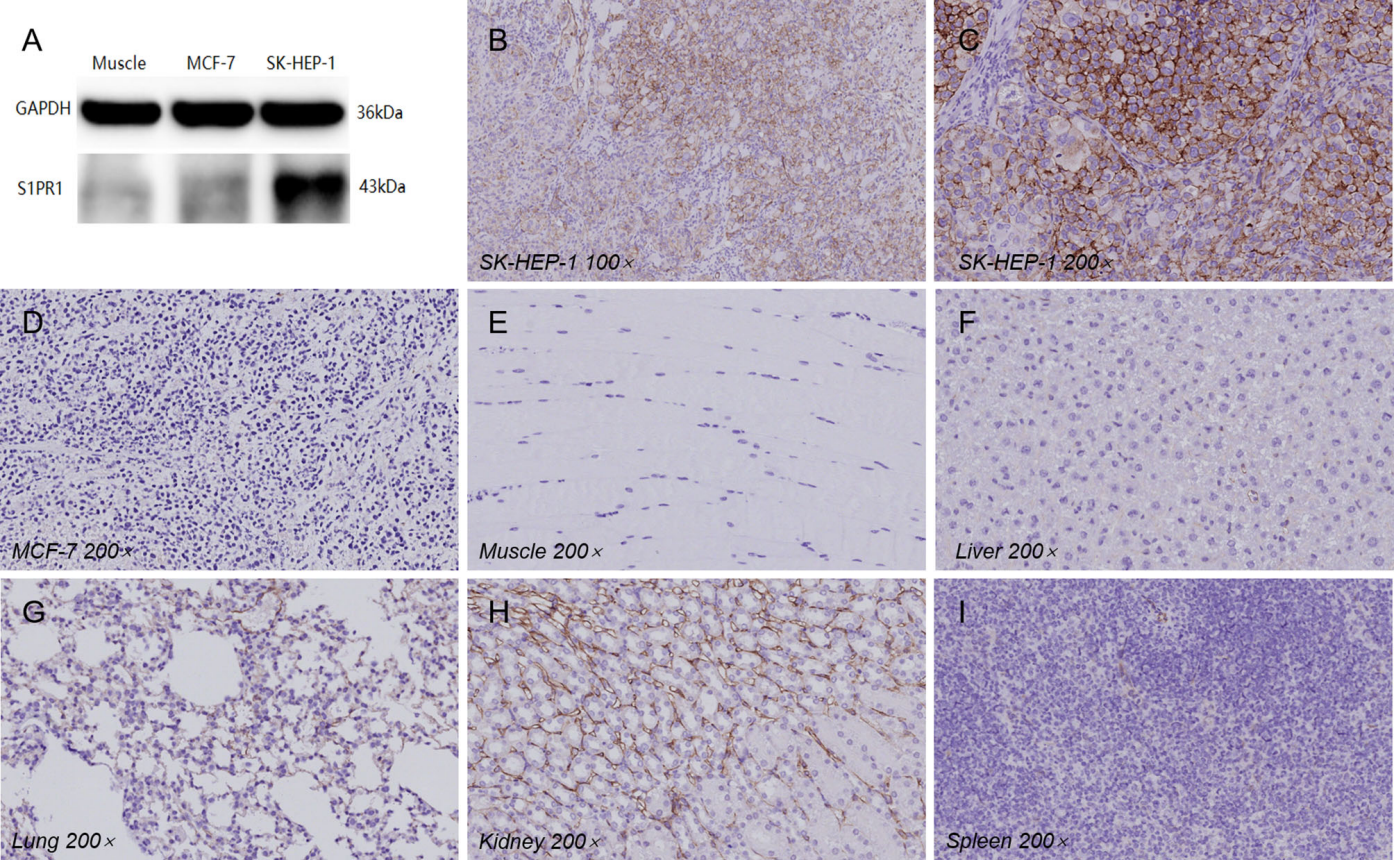
**(A)** Western blot analysis of S1PR1 expression in SK-HEP-1, MCF-7 tumor tissues and muscle. **(B–I)** The immunohistochemical staining results of various organs and tissues (under 100× and 200× magnification).

Western blotting confirmed that S1PR1 is highly expressed in SK-HEP-1 solid tumors, while the expression of S1PR1 in muscle tissues from nude mice in the experimental group and MCF-7 tumor tissues is very low (**Figure 4A**). Immunohistochemistry staining confirmed that a small amount of S1PR1 was expressed in vascular endothelial cells in lung, liver, spleen, kidney, muscle and MCF-7 tumor tissues, while the cell membranes of SK-HEP-1 tumors highly expressed S1PR1 (**Figures 4B–I**).

## Discussion

We developed a molecular probe, ^99m^Tc-HYNIC-S1PR1mAb, that can bind to S1PR1 both *in vitro* and *in vivo.* The uptake rate of ^99m^Tc-HYNIC-S1PR1mAb in tumor cells was related to the expression of S1PR1 and could be specifically blocked. The specificity of ^99m^Tc-HYNIC-S1PR1mAb to S1PR1 was verified at the cellular level. The uptake rate of ^99m^Tc-HYNIC-S1PR1mAb in SK-HEP-1 cells (S1PR1 high expression) was more than 2-fold greater than in MCF-7 (S1PR1 low expression) or blocking groups.

SPECT imaging and biodistribution results show that tumors with high expression of S1PR1 can specifically take up ^99m^Tc-HYNIC-S1PR1mAb. The tumor uptake in the blocking group and the MCF-7 xenografted mice group were close to the background level at 2 h and were significantly lower than in the SK-HEP-1 group at 2 h. The results of SPECT imaging were consistent with the biodistribution study results. At 24 h, the tumor uptake value of the SK-HEP-1 group was 5.53 ± 0.32% ID/g, which was higher than both blocking group (3.64 ± 0.39% ID/g, P = 0.003) and MCF-7 group (2.81 ± 0.21% ID/g, P = 0.000), and indicated that the molecular probe had targeting potential and specificity for tumors with high S1PR1 expression. The tumor/blood ratio and tumor/muscle ratio of the SK-HEP-1 group were 0.39 ± 0.01 and 5.99 ± 1.38, and the tumor/muscle ratio was more than twice that of the blocking group (2.84 ± 0.52, P = 0.020) and the MCF-7 group (2.38 ± 0.07, P = 0.011). The results indicated that ^99m^Tc-HYNIC-S1PR1mAb has a good targeting ability to S1PR1 and could be used as an imaging tracer for S1PR1.

SPECT imaging showed that ^99m^Tc-HYNIC-S1PR1mAb was mainly distributed in the liver, and the tumor in the experimental group was not obvious at 2 h. Subsequently, the imaging agent at the tumor site gradually concentrated with time, and the tumor was seen vaguely at 12 h, and the tumor was clearly seen at 18 h and 24 h. ^99m^Tc-HYNIC-S1PR1mAb is retained in the blood and non-target organs, especially in the liver, spleen and kidneys. However, the immunohistochemistry result of tissues showed that S1PR1 only had a mild expression in the liver. The high uptake of the tracer might be related to the metabolism of ^99m^Tc-HYNIC-S1PR1mAb through the liver, which is similar to the results of other experiments using monoclonal antibodies as radioactive probes (24–26). Antigen-antibody complexes will be non-specifically taken up by the reticuloendothelial system of the liver and spleen, resulting in a concentration of radioactivity. This retention characteristic has a great impact on the evaluation of liver tumors. The high activity in the kidney can be attributed to the renal clearance of radioactive degradation products (27), so it is necessary to further improve the stability of molecular probes *in vivo*. In addition, the FTY720 may have anti-cancer effects, so the reducing reduction of antibodies may be due to the decrease in tumor, rather than decreased S1PR1. However, in our studies, we usually did not collect the whole tumor tissues in biodistribution to analyze. The valid data of 24 cases (complete tumor was collected), including 12 cases in the experimental group and 12 cases in the blocking group showed that the difference in tumor volume between the two groups was not statistically significant (P>0.05). FTY720 does have the potential to kill tumors, but this is not reflected in our data, probably because our sample size is limited. However, we will explore more blocking conditions or try different blockers (such as the blocking of the antibody itself) in future experiments.

S1PR1 has played an important role in tumor, inflammation, immune system, vascular regulation, etc. (11–13). It could be a key biomarker in immune responses, inflammation, and in the prognosis of certain tumors (4, 28). Current research focuses on the synthesis of small molecule radioactive probes binding to S1PRs for PET scanning. For example, ^11^C-TZ3321 can detect the up-regulation of S1PR1 in inflammatory blood vessels, and the expression of S1PR1 is higher in neointimal hyperplasia, so ^11^C-TZ3321 may realize the detection of early inflammation or can be used in the diagnosis and prognosis of atherosclerosis (29, 30). To extend the imaging time, some studies have used ^18^F-labeled S1PR1 radioligands. After the molecular probes traversed the blood–brain barrier, an *in vivo* autoradiographic study of the neuroinflammatory response in mice was performed, and S1PR1 imaging in primates was achieved with satisfactory results (31, 32). Despite the challenges and drawbacks, newer ^99m^Tc-based S1PR1-targeted tracers should also be studied, for SPECT imaging is widely used.

Although the use of radiolabeled monoclonal antibodies can achieve a good target-to-background ratio, the longer circulating half-life is the main limitation of using them as molecular imaging probes (33). For example, the tumor in this experiment was clearly visible at 24 hours, but the image quality obtained after 4 half-lives of the nuclide is not good enough. Longer-lived isotopes may be a better choice, such as indium-111. To optimize the target-to-background ratio, pretargeting techniques may be used in the future. The monoclonal antibody and the small molecule radioligand are injected successively. When the radioligand is injected, the antibody has accumulated in the tumor and most unbound antibody has already been removed from the bloodstream. Pre-targeting techniques can significantly reduce the uptake of tracer in non-target tissues, which is beneficial to achieve the purpose of radioimmunoimaging using short half-life nuclides or large molecular weight monoclonal antibodies (34, 35).

In addition, to optimize the imaging results, small molecule probes aimed at the same target can be selected, such as small molecule radioligands, targeting peptides, Fab fragments (36). Targeting peptides have a shorter circulating half-life, better permeability, lower immunogenicity, and are easily chemically modified, so they may be more suitable for scientific research and clinical imaging (37–40). Li used ^99m^Tc-labeled HER2-targeted peptides for experiments, and the results showed that the maximum absorption value of the was observed 30 min after injection of the imaging agent, and the highest tumor/organ radioactive uptake ratio was observed within 1 h (36). Fab fragments retain the same specificity and affinity activity as monoclonal antibodies. Compared with intact antibodies, Fab fragments have a smaller molecular weight and faster metabolism in the body. The lack of Fc fragments can reduce non-specific uptake. Clear tumor visualization and high tumor/background signal ratio can be obtained in a short time (33, 41). In general, the reduction in molecular weight optimizes the imaging performance of the probe, and small molecule probes can be combined with positron nuclides with a short half-life to perform rapid PET imaging. The sensitivity and spatial resolution of PET are higher than that of SPECT, which can provide more accurate information for the study of S1PR1 and the diagnosis and treatment related to S1PR1. This will be one of the development directions of radioimmunoimaging in the future.

## Conclusion

We developed a molecular probe, ^99m^Tc-HYNIC-S1PR1mAb, that can target to S1PR1. Both *in vitro* and *in vivo* experiments showed that ^99m^Tc-HYNIC-S1PR1mAb have good specificity and affinity. Further studies could be performed to optimize the probe for better pharmacokinetics *in vivo.*

## Data Availability Statement

The original contributions presented in the study are included in the article/**Supplementary Material**. Further inquiries can be directed to the corresponding authors.

## Ethics Statement

The animal study was reviewed and approved by Institutional Animal Care and Use Committee of Tongji Medical College, Huazhong University of Science and Technology.

## Author Contributions

XX and XL designed the research. MY, YG, HJ, YJ, PQ, and WW performed research. MY analysed the data. MY and XX wrote the paper. YG, YZ, and XL reviewed the paper. All authors contributed to the article and approved the submitted version.

## Funding

This research was supported by National Natural Science Foundation of China (No. 81801737), Fundamental research fund for the Chinese Central Universities of Huazhong University of Science and Technology (HUST) (2017KFYXJJ246) and China Scholarship Council (CSC) Visiting Scholar Grant (No. 201906165056) to XX.

## Conflict of Interest

The authors declare that the research was conducted in the absence of any commercial or financial relationships that could be construed as a potential conflict of interest.

## Publisher’s Note

All claims expressed in this article are solely those of the authors and do not necessarily represent those of their affiliated organizations, or those of the publisher, the editors and the reviewers. Any product that may be evaluated in this article, or claim that may be made by its manufacturer, is not guaranteed or endorsed by the publisher.

## References

[1] Yanagida K Hla T. Vascular and Immunobiology of the Circulatory Sphingosine 1-Phosphate Gradient. Annu Rev Physiol (2017) 79:67–91. 10.1146/annurev-physiol-021014-071635 27813829 PMC5500220

[2] Liu Y Zhi Y Song H Zong M Yi J Mao G. S1PR1 Promotes Proliferation and Inhibits Apoptosis of Esophageal Squamous Cell Carcinoma Through Activating STAT3 Pathway. J Exp Clin Cancer Res (2019) 38:369. 10.1186/s13046-019-1369-7 31438989 PMC6706905

[3] Rodriguez YI Campos LE Castro MG Aladhami A Oskeritzian CA Alvarez SE. Sphingosine-1 Phosphate: A New Modulator of Immune Plasticity in the Tumor Microenvironment. Front Oncol (2016) 6:218. 10.3389/fonc.2016.00218 27800303 PMC5066089

[4] Kunkel GT Maceyka M Milstien S Spiegel S. Targeting the Sphingosine-1-Phosphate Axis in Cancer, Inflammation and Beyond. Nat Rev Drug Discov (2013) 12:688–702. 10.1038/nrd4099 23954895 PMC3908769

[5] Rosen H Stevens RC Hanson M Roberts E Oldstone MB. Sphingosine-1-Phosphate and Its Receptors: Structure, Signaling, and Influence. Annu Rev Biochem (2013) 82:637–62. 10.1146/annurev-biochem-062411-130916 23527695

[6] Li MH Sanchez T Yamase H Hla T Oo ML Pappalardo A. S1P/S1P1 Signaling Stimulates Cell Migration and Invasion in Wilms Tumor. Cancer Lett (2009) 276:171–9. 10.1016/j.canlet.2008.11.025 PMC2943759 19131156

[7] Young N Pearl DK Van Brocklyn JR. Sphingosine-1-Phosphate Regulates Glioblastoma Cell Invasiveness Through the Urokinase Plasminogen Activator System and CCN1/Cyr61. Mol Cancer Res (2009) 7:23–32. 10.1158/1541-7786.MCR-08-0061 19147534 PMC2708075

[8] Lin Q Wei Y Zhong Y Zhu D Ren L Xu P. Aberrant Expression of Sphingosine-1-Phosphate Receptor 1 Correlates With Metachronous Liver Metastasis and Poor Prognosis in Colorectal Cancer. Tumour Biol (2014) 35:9743–50. 10.1007/s13277-014-2267-4 24972972

[9] Watson C Long JS Orange C Tannahill CL Mallon E McGlynn LM. High Expression of Sphingosine 1-Phosphate Receptors, S1P1 and S1P3, Sphingosine Kinase 1, and Extracellular Signal-Regulated Kinase-1/2 Is Associated With Development of Tamoxifen Resistance in Estrogen Receptor-Positive Breast Cancer Patients. Am J Pathol (2010) 177:2205–15. 10.2353/ajpath.2010.100220 PMC2966780 20889557

[10] Liu Y-N Zhang H Zhang L Cai T-T Huang D-J He J. Sphingosine 1 Phosphate Receptor-1 (S1P1) Promotes Tumor-Associated Regulatory T Cell Expansion: Leading to Poor Survival in Bladder Cancer. Cell Death Dis (2019) 10:50. 10.1038/s41419-018-1298-y 30718502 PMC6362099

[11] Odate S Veschi V Yan S Lam N Woessner R Thiele CJ. Inhibition of STAT3 With the Generation 2.5 Antisense Oligonucleotide, AZD9150, Decreases Neuroblastoma Tumorigenicity and Increases Chemosensitivity. Clin Cancer Res (2017) 23:1771–84. 10.1158/1078-0432.CCR-16-1317 PMC5381521 27797972

[12] Sarkisyan G Gay LJ Nguyen N Felding BH Rosen H. Host Endothelial S1PR1 Regulation of Vascular Permeability Modulates Tumor Growth. Am J Physiol Cell Physiol (2014) 307:C14–24. 10.1152/ajpcell.00043.2014 PMC4080184 24740542

[13] Chae SS Paik JH Furneaux H Hla T. Requirement for Sphingosine 1-Phosphate Receptor-1 in Tumor Angiogenesis Demonstrated by *In Vivo* RNA Interference. J Clin Invest (2004) 114:1082–9. 10.1172/JCI200422716 PMC522258 15489955

[14] Gerald D Chintharlapalli S Augustin HG Benjamin LE. Angiopoietin-2: An Attractive Target for Improved Antiangiogenic Tumor Therapy. Cancer Res (2013) 73:1649–57. 10.1158/0008-5472.CAN-12-4697 23467610

[15] Fischl AS Wang X Falcon BL Almonte-Baldonado R Bodenmiller D Evans G. Inhibition of Sphingosine Phosphate Receptor 1 Signaling Enhances the Efficacy of VEGF Receptor Inhibition. Mol Cancer Ther (2019) 18:856–67. 10.1158/1535-7163.MCT-18-0548 PMC6992361 30787172

[16] Prasad VP Wagner S Keul P Hermann S Levkau B Schäfers M. Synthesis of Fluorinated Analogues of Sphingosine-1-Phosphate Antagonists as Potential Radiotracers for Molecular Imaging Using Positron Emission Tomography. Bioorg Med Chem (2014) 22:5168–81. 10.1016/j.bmc.2014.08.009 25216968

[17] Liu H Jin H Yue X Luo Z Liu C Rosenberg AJ. PET Imaging Study of S1PR1 Expression in a Rat Model of Multiple Sclerosis. Mol Imaging Biol (2016) 18:724–32. 10.1007/s11307-016-0944-y PMC5297893 26975859

[18] Rosenberg AJ Liu H Jin H Yue X Riley S Brown SJ. Design, Synthesis, and In Vitro and In Vivo Evaluation of an (18)F-Labeled Sphingosine 1-Phosphate Receptor 1 (S1P1) PET Tracer. J Med Chem (2016) 59:6201–20. 10.1021/acs.jmedchem.6b00390 PMC5091660 27280499

[19] Schilson SS Keul P Shaikh RS Schäfers M Levkau B Haufe G. Synthesis of New Ligands for Targeting the S1P1 Receptor. Bioorg Med Chem (2015) 23:1011–26. 10.1016/j.bmc.2015.01.014 25656338

[20] Luo Z Liu H Klein RS Tu Z. Design, Synthesis, and *In Vitro* Bioactivity Evaluation of Fluorine-Containing Analogues for Sphingosine-1-Phosphate 2 Receptor. Bioorg Med Chem (2019) 27:3619–31. 10.1016/j.bmc.2019.06.047 PMC6698139 31279524

[21] Rokka J Federico C Jurttila J Snellman A Haaparanta M Rinne JO. 19F/18F Exchange Synthesis for a Novel 18F]S1P3-Radiopharmaceutical. J Labelled Comp Radiopharm (2013) 56:385–91. 10.1002/jlcr.3055 24285478

[22] Rosenberg AJ Liu H Tu Z. A Practical Process for the Preparation of [(32)P]S1P and Binding Assay for S1P Receptor Ligands. Appl Radiat Isot (2015) 102:5–9. 10.1016/j.apradiso.2015.04.010 25931137 PMC4457593

[23] Liu H Jin H Han J Yue X Yang H Zayed MA. Upregulated Sphingosine 1-Phosphate Receptor 1 Expression in Human and Murine Atherosclerotic Plaques. Mol Imaging Biol (2018) 20:448–56. 10.1007/s11307-017-1141-3 PMC5975257 29134505

[24] Calzada V Garcia F Fernández M Porcal W Quinn T Alonso O. Labeling and Biological Evaluation of (99m)Tc-HYNIC-Trastuzumab as a Potential Radiopharmaceutical for In Vivo Evaluation of HER2 Expression in Breast Cancer. World J Nucl Med (2013) 12:27–32. 10.4103/1450-1147.113953 23961253 PMC3745630

[25] Li D Cheng S Zou S Zhu D Zhu T Wang P. Immuno-PET Imaging of Zr Labeled Anti-PD-L1 Domain Antibody. Mol Pharm (2018) 15:1674–81. 10.1021/acs.molpharmaceut.8b00062 29502426

[26] Brumley CL Kuhn JA. Radiolabeled Monoclonal Antibodies. AORN J (1995) 62343–50. 10.1016/S0001-2092(06)63575-8 8534053

[27] Carpenet H Cuvillier A Monteil J Quelven I. Anti-CD20 Immunoglobulin G Radiolabeling With a 99mtc-Tricarbonyl Core: *In Vitro* and *In Vivo* Evaluations. PLoS One (2015) 10:e0139835. 10.1371/journal.pone.0139835 26439852 PMC4595339

[28] Lifshitz V Priceman SJ Li W Cherryholmes G Lee H Makovski-Silverstein A. Sphingosine-1-Phosphate Receptor-1 Promotes Environment-Mediated and Acquired Chemoresistance. Mol Cancer Ther (2017) 16:2516–27. 10.1158/1535-7163.MCT-17-0379 PMC5669816 28716816

[29] Jin H Yang H Liu H Zhang Y Zhang X Rosenberg AJ. A Promising Carbon-11-Labeled Sphingosine-1-Phosphate Receptor 1-Specific PET Tracer for Imaging Vascular Injury. J Nucl Cardiol (2017) 24:558–70. 10.1007/s12350-015-0391-1 26843200

[30] Liu H Jin H Yue X Han J Baum P Abendschein DR. PET Study of Sphingosine-1-Phosphate Receptor 1 Expression in Response to Vascular Inflammation in a Rat Model of Carotid Injury. Mol Imaging (2017) 16:1536012116689770. 10.1177/1536012116689770 28654378 PMC5470136

[31] Liu H Luo Z Gu J Jiang H Joshi S Shoghi KI. In Vivo Characterization of Four F-Labeled S1PR1 Tracers for Neuroinflammation. Mol Imaging Biol (2020) 22:1362–9. 10.1007/s11307-020-01514-8 PMC7679043 32602083

[32] Luo Z Rosenberg AJ Liu H Han J Tu Z. Syntheses and *In Vitro* Evaluation of New S1PR1 Compounds and Initial Evaluation of a Lead F-18 Radiotracer in Rodents. Eur J Med Chem (2018) 150:796–808. 10.1016/j.ejmech.2018.03.035 29604582 PMC5908474

[33] Mendler CT Gehring T Wester H-J Schwaiger M Skerra A. ^8 9^ Zr-Labeled Versus ^1 2 4^ I-Labeled αher2 Fab With Optimized Plasma Half-Life for High-Contrast Tumor Imaging In Vivo. J Nucl Med (2015) 56:1112–8. 10.2967/jnumed.114.149690 25999431

[34] van de Watering FCJ Rijpkema M Robillard M Oyen WJG Boerman OC. Pretargeted Imaging and Radioimmunotherapy of Cancer Using Antibodies and Bioorthogonal Chemistry. Front Med (Lausanne) (2014) 1:44. 10.3389/fmed.2014.00044 25593917 PMC4292049

[35] Keinänen O Fung K Pourat J Jallinoja V Vivier D Pillarsetty NK. Pretargeting of Internalizing Trastuzumab and Cetuximab With a F-Tetrazine Tracer in Xenograft Models. EJNMMI Res (2017) 7:95. 10.1186/s13550-017-0344-6 29198065 PMC5712296

[36] Li L Wu Y Wang Z Jia B Hu Z Dong C. SPECT/CT Imaging of the Novel HER2-Targeted Peptide Probe Tc-HYNIC-H6F in Breast Cancer Mouse Models. J Nucl Med (2017) 58:821–6. 10.2967/jnumed.116.183863 28104744

[37] Deutscher SL. Phage Display in Molecular Imaging and Diagnosis of Cancer. Chem Rev (2010) 110:3196–211. 10.1021/cr900317f PMC2868952 20170129

[38] Lee H Deng J Kujawski M Yang C Liu Y Herrmann A. STAT3-Induced S1PR1 Expression Is Crucial for Persistent STAT3 Activation in Tumors. Nat Med (2010) 16:1421–8. 10.1038/nm.2250 PMC3088498 21102457

[39] Li H Yuan L Long Y Fang H Li M Liu Q. Synthesis and Preclinical Evaluation of a Ga-Radiolabeled Peptide Targeting Very Late Antigen-3 for PET Imaging of Pancreatic Cancer. Mol Pharm (2020) 17:3000–8. 10.1021/acs.molpharmaceut.0c00416 32544337

[40] Gai Y Jiang Y Long Y Sun L Liu Q Qin C. Evaluation of an Integrin αβ and Aminopeptidase N Dual-Receptor Targeting Tracer for Breast Cancer Imaging. Mol Pharm (2020) 17:349–58. 10.1021/acs.molpharmaceut.9b01134 PMC7486978 31829615

[41] Chakravarty R Goel S Valdovinos HF Hernandez R Hong H Nickles RJ. Matching the Decay Half-Life With the Biological Half-Life: ImmunoPET Imaging With (44)Sc-Labeled Cetuximab Fab Fragment. Bioconjug Chem (2014) 25:2197–204. 10.1021/bc500415x PMC4275156 25389697

