# Supplementary material for: A Novel Radioimmune 99mTc-Labeled Tracer for Imaging Sphingosine 1-Phosphate Receptor 1 in Tumor Xenografts: An In Vitro and In Vivo Study
Source: Front Immunol. 2021 Aug 18;12:660842. doi: 10.3389/fimmu.2021.660842 (PMC8416251; doi:10.3389/fimmu.2021.660842)
Supplement: Supplementary file 1 [file DataSheet_1.docx]

Supplementary Material

# Supplementary Data

## The quality control results of antibody labeling and purification

## The labeled compound was purified on a PD-10 column (General Electric, Milwaukee WI, USA). We used the liquid collected from the 2/3/4 tubes with the highest radioactivity (500 μL/tube). The radiolabeled compound was analyzed by instant thin layer chromatography (ITLC) to calculate its radiochemical purity (Supplementary Figure 1). After purification on a PD-10 column, the radiochemical purity was 96.7 ± 0.04% (n=4).


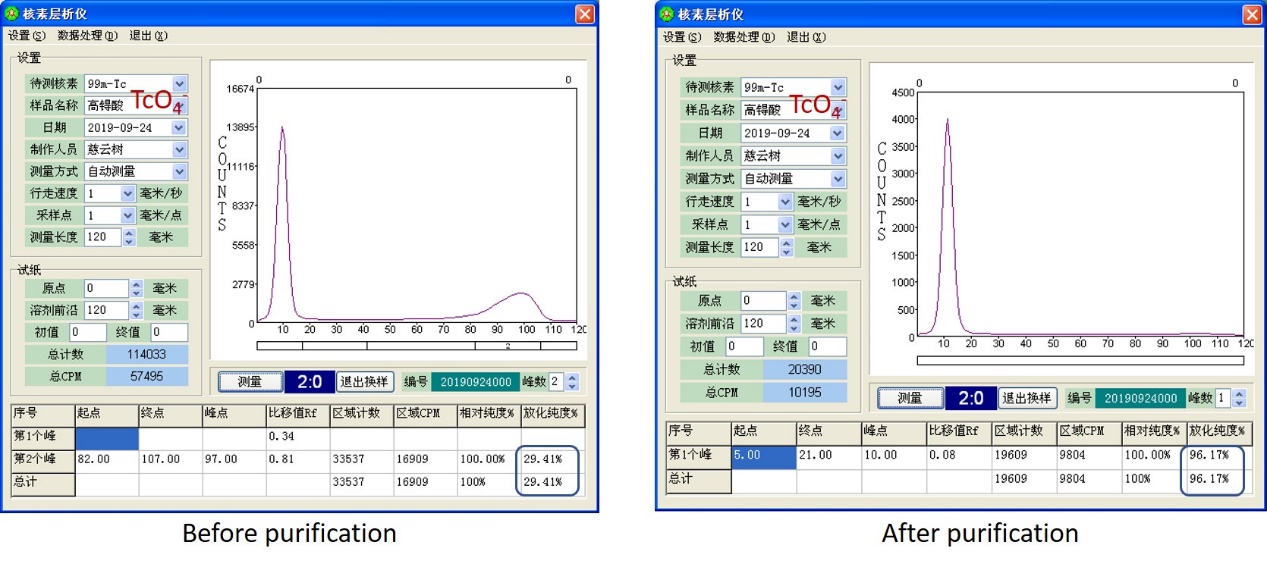


Supplementary Figure 1 The thin layer chromatography of ^99m^Tc-HYNIC-S1PR1mAb

## Quantitative real-time PCR (qRT-PCR)

Total RNA was extracted from SK-HEP-1, Jurkat and MCF-7 cell lines using the TRIzol reagent (Takara, Japan), according to the manufacturer’s guidelines. RNAs were reverse transcribed using the SYBR-Green super mix (cat. no. RR036A; Takara, Japan) according to the manufacturer’s instructions. Then, qRT-PCR was conducted using the SYBR Green reagent (Cat no. RR420A; TaKaRa, Japan) according to the manufacturer’s instructions. Human GAPDH was utilized as the internal control. The relative level of each mRNA was calculated using the 2−ΔΔC method.

In the introduction of antibodies on Abcam(ab233386), Jurkat cells are mentioned as positive control of S1PR1, so we perform qRT-PCR on these cells together. The Jurkat cell line were purchased from the Type Culture Collection of the Chinese Academy of Sciences, Shanghai, China (CAT# TCHU123).

The results were as follows: the expression level of S1PR1 mRNA in SK-HEP-1 cell line was about 9 times that of McF-7 cell line (Table 1-2, Supplementary Figure 2-3).

| gene name | Sample Name | Ct | GAPDH Ct | Mean GAPDH Ct | △Ct | Mean △Ct | -ΔΔCt | Expression(2-ΔΔCt) |
| --- | --- | --- | --- | --- | --- | --- | --- | --- |
| S1PR1 | MCF-7 | 27.35 | 13.25 | 13.37 | 13.98 | 13.97333333 | -0.01 | 0.993092495 |
| S1PR1 | MCF-7 | 27.27 | 13.44 | 13.37 | 13.9 |  | 0.07 | 1.049716684 |
| S1PR1 | MCF-7 | 27.41 | 13.41 | 13.37 | 14.04 |  | -0.07 | 0.952637998 |
| S1PR1 | jurkat | 26.98 | 13.5 | 13.68 | 13.3 |  | 0.67 | 1.591072968 |
| S1PR1 | jurkat | 24.78 | 13.8 | 13.68 | 11.1 |  | 2.87 | 7.310651602 |
| S1PR1 | jurkat | 25.57 | 13.75 | 13.68 | 11.89 |  | 2.08 | 4.228072162 |
| S1PR1 | SK-HEP-1 | 24.55 | 13.85 | 13.79 | 10.76 |  | 3.21 | 9.253505471 |
| S1PR1 | SK-HEP-2 | 24.5 | 13.77 | 13.79 | 10.71 |  | 3.26 | 9.579829637 |
| S1PR1 | SK-HEP-3 | 24.8 | 13.76 | 13.79 | 11.01 |  | 2.96 | 7.781239579 |

Table 1 Quantitative real-time PCR table 1

Table 2 Quantitative real-time PCR table 2

| gene name | Sample Name | Mean Expression(2-ΔΔCt) | STDEV |
| --- | --- | --- | --- |
| S1PR1 | MCF-7 | 0.998482392 | 0.048763265 |
| S1PR1 | jurkat | 4.376598911 | 2.862680577 |
| S1PR1 | SK-HEP-1 | 8.871524896 | 0.958208442 |

Supplementary Figure 2 The expression of S1PR1 mRNA of MCF-7, Jurkat and SK-HEP-1 cell.


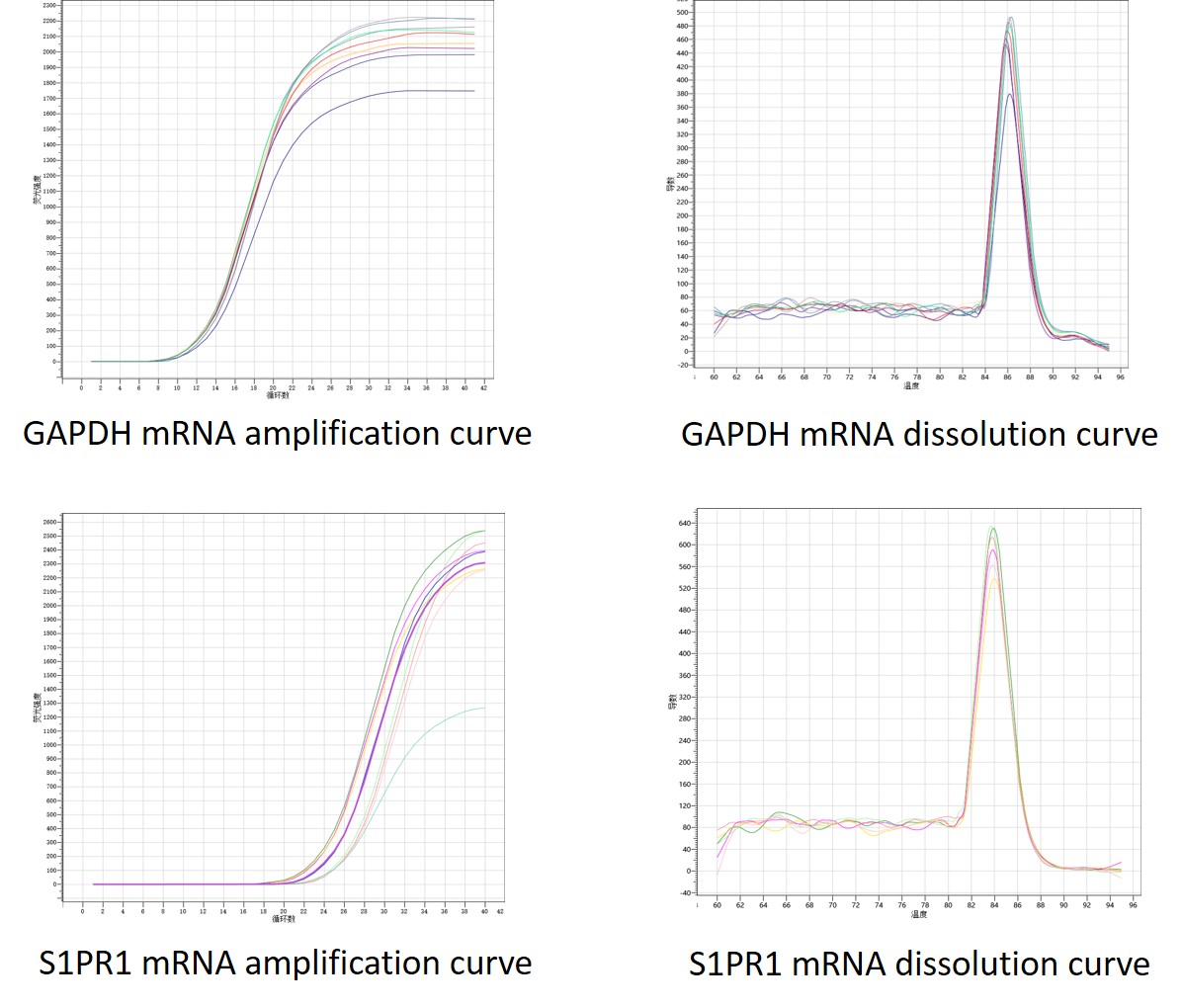


Supplementary Figure 3 The quantitative real-time PCR of MCF-7, Jurkat and SK-HEP-1 cell.

## Western Blot

When SK-HEP-1 and MCF-7 cells grew to a density of about 80%, we collected them and added cell lysate buffer (Beyotime, Shanghai, China) and isolated the protein. The protein concentration was determined using a bicinchoninic acid (BCA) protein assay kit (Boster, Wuhan, China). After SDS-PAGE electrophoresis, proteins were transferred to PVDF membrane. The membrane was incubated with the anti-S1PR1 antibody (Abcam, ab233386) for one night and then an anti-rabbit secondary antibody (Sanjian, Tianjin, China, LK2001) for 2 h. Images were processed with Image J software. Results are as follows. The Supplementary Figure 4 showed the repeated result of Western blot analysis (with uncropped file) of S1PR1 expression in SK-HEP-1 cells and MCF-7 cells.


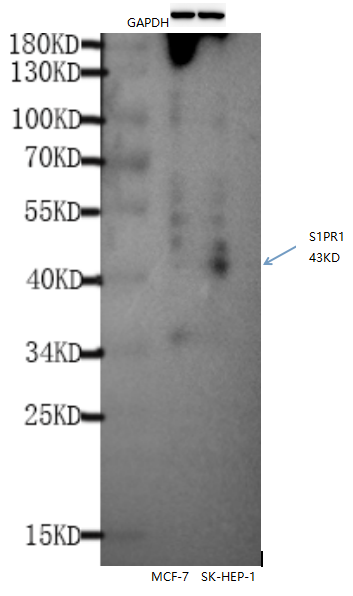


Supplementary Figure 4 Western blot analysis (with uncropped file) of S1PR1 expression in SK-HEP-1 cells and MCF-7 cells.
